# Vermicomposting and anaerobic digestion – viable alternative options for terrestrial weed management – A review

**DOI:** 10.1016/j.btre.2017.11.005

**Published:** 2017-11-22

**Authors:** Biswanath Saha, Chaichi Devi, Meena Khwairakpam, Ajay S. Kalamdhad

**Affiliations:** a Centre for the Rural Technology, Indian Institute of Technology, Guwahati 781039, Assam, India; b Department of Civil Engineering, National Institute of Technology, Meghalaya 793003, India; c Centre for the Rural Technology, Indian institute of Technology, Guwahati 789039, Assam, India; d Department of Civil Engineering, Indian Institute of Technology, Guwahati 789039, Assam, India

**Keywords:** Terrestrial weeds, Vermicomposting, Anaerobic digestion, Allelopathy, Biogas

## Abstract

The management of terrestrial weed is of great concern for the scientific community as these weeds cause adverse effect in different ecosystems like forest, agriculture and urban. The widespread of these weeds by their adaptive capability and morphological advancement is difficult to control. *Parthenium hysterophorous*, *Lantana camara*, *Saccharum spontaneum, Ageratum conyzoides* are the weeds that spread all over the world. There are various management practices employed for the control of this weeds. But all of these practices have some drawbacks those are neither environment friendly nor economical. In this paper a review has been done to evaluate various alternative management practices for these terrestrial weeds and to analyze their feasibility. Vermicomposting and anaerobic digestion can be viable alternative option which is cost effective as well. There are few studies regarding vermicomposting and anaerobic digestions of terrestrial weeds are done.

## Highlights

• Management of terrestrial weeds is one of the key concerns in world wide.
• These noxious weeds participate with crops with their natural resources.
• Terrestrial weeds can be managed by anaerobic digestion and vermicomposting.
• Most of these weeds contain high lignin.
• Pretreatment is required to further recovery of energy.

## 1 Introduction

A wild plant growing where it is not required and is competition with cultivated plant is termed as weed. There is a dynamic system that involves the interaction between weeds, crops, humans and environment. According to different definitions of weed – a plant growing where it is not wanted, a plant out of place, a plant growing where it is desired that something else growth, those plants with harmful or objectionable habits. Therefore, it can be summarized that weeds are those uninvited plants which are grown in undesirable place and period causing competition for cultivated crops and economic loss. Invasive plant species not only change the dynamics of species composition and biodiversity but also hamper the system productivity and efficiency in invaded regions [1]. Besides rapidly colonizing areas replacing the native vegetation, it is also known to cause a number of human health problems, environmental degradation including threat to tourism activities [2]. The characteristics that make a plant weed are- (1) long seed life in the soil, (2) quick mergence, (3) rapid early growth, (4) no special environmental requirements for germination, (5) ability to survive and prosper under disturbed condition. Organizations engaged in invasion research defined invasive species as a species that is not native to the ecosystem under consideration whose introduction causes or is likely to cause economic or environmental harm or harm to human health, species, subspecies or lower taxon, introduced outside its natural past or present distribution; includes any part, gametes, seeds, eggs, or propagates of such species that might survive and subsequently reproduce, animals, plants or other organisms introduced by man into places out of their natural range of distribution, where they become established and disperse, generating a negative impact on the local ecosystem and species, an alien species whose introduction does or is likely to cause economic or environmental harm or harm to human health (Invasive Species Advisory Council, 2001). In India, exotic weeds, especially *Parthenium hysterophorus* in urban areas*, Lantana camara* in forestlands, and *Ageratum conyzoides* in croplands, have assumed the proportion of noxious biological pollutants. Each of these three natives of tropical America has wide ecological amplitude. Because of ecological, agricultural, environmental, and health hazards for cattle and man, the respective governments of the states as well as the union government of India are trying hard to assess the damage and find a solution for their control [3]. Twelve sustainable weed management strategies are described as [4]: Know the weeds on your farm, Plant cropping systems to minimize open niches to weeds, keep the weed guessing, design the cropping system and select tools for effective weed control, grow vigorous, competitive crops, put the weeds out of work-grow cover crops, manage the weed seed bank, minimize deposition and maximize withdrawals, Know the weeds out at critical time, utilize biological processes to enhance weed control, bring existing weeds under control before planting sensitive crops and long term perennial crops, keep observing the weeds and adapt practices accordingly. In India, *Parthenium* it was testified that it cause 40% damages in yield of agricultural crops [5] whereas in Australia, an yearly damage of $16.8 million to the cattle industry was valued due to presence of this weed in grasslands [6]. Crop likes black gram (*P. mungo*), throughout first 30–45 days after crop planting, the existence of this weed caused significant yield losses, therefore management of this weed is essential [7]. Keep up on new development and practices have been done but for the developing countries, the use of organic manure on crop will not only improve the soil properties but will also cut down on the foreign exchange need for the purchase of mineral fertilizers. Weeds are available plenty but they are not wanted [8]. Weeds are unwanted plants but which can be converted into valuable resources and available free of cost growing without cultivation, irrigation and protecting the soil by giving of a warm soil cover. A farmer can produce his individual vermicompost from the biodegradable waste like weeds, made their own farm and need not spend extra money to purchase the raw material of vermicompost. Chemical fertilizers have been one of the major components of modern agriculture. Use of chemicals has now raised many questions related to the productivity of land and continuously increasing cost of cultivation. Vermicompost technology has been solved many problems. Earthworms have been known as farmer’s friends for long (Darwin, 1881). Vermicompost technology is converting all biodegradable waste into plant nutrient rich organic manure with the help of composting.

## 2 Allelopathy

Allelopathy is an important mechanism of plant interference by the addition of plant-produced phytotoxins to the environment. Many of the phototoxic substance suspected of causing germination and growth inhibition have been identified from plant tissues and soil, these substances are termed allelochemics or allelochemicals. Allelochemistry, the production and release of toxic chemicals produced by one species that affect a receiving susceptible species, has been the subject of diverse degrees of scientific enquiry. Recent advances in plant biology have permitted the revamp of allelochemistry as a biologically and ecologically sound explanation for plant invasion and plant–plant communication in the rhizosphere. Recent progress has been made in understanding the biochemical and molecular changes that are induced by allelochemicals in susceptible plant species, and the complex mechanisms that are used by allelochemical-resistant plants to defend against this toxic insult [9].

### 2.1 Terrestrial weeds

Terrestrial weeds which are evident in India and many other parts of the world causing economic and environmental threats are *Lantana camara, Ageratum conyzoides, Parthenium hysterophorus, Galinsoga purviflora, Saccharum spontaneum, Argemone Mexicana*.

#### 2.1.1 Lantana camara

**Fig. 1:**
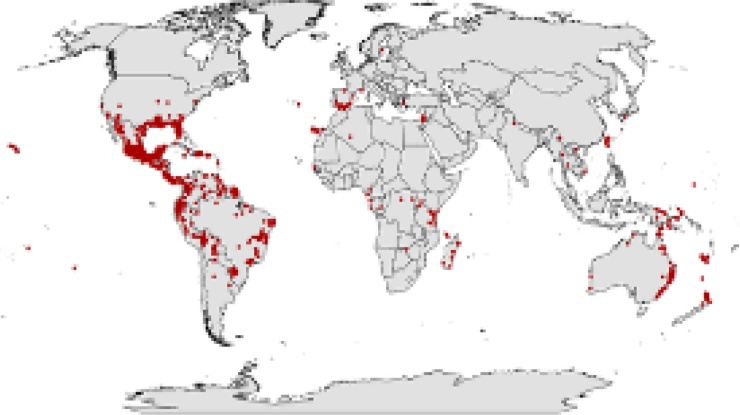
The current global spreading of *L. c a m a r a* taken from the Global Biodiversity Information Facility 2007. Red dots show existence records of *L. camara*.

*Lantana camara* is the one of the ten worst weeds of the world belonging to the family Verbenaceae. Lantana forms huge dense thickets on the floor of forest and plantation areas due to its scrambling growth patterns and bushy nature which hampers normal function of the forest and the growth and development of other underlying vegetation (Iyenger 1933; Singh et al., 1988) as reported in Fig. 1. Native of Central and South America, it is an evergreen aromatic shrub [10]. In India it was introduced during 1809–1810 as an ornamental plant in Calcutta’s gardens [11]. Now all over India this weed is commonly found. More than 13.2 million ha pasture land and other areas in India had been invaded by *Lantana* [12]. This weed has replaced *Quercus leucotrichphora* and *Pinus roxburghii* forests in Kumaun hills (U.P.) [13]; invaded the Teak plantations in Tamil Nadu; covered Western Ghats (South India) [15] and heart water region of Garhwal (U.P.) [16]. The cost of *Lantana* management is US$ 70 per hectare and is harmful to herbivores [12]. The soil of *Lantana* invaded and non-invaded when analysed edaphic factors such as soil pH, total nitrogen, soil organic carbon, phosphorus and potassium content positively influenced the growth of *Lantana* and helped in the further invasion process. *Lantana* invasion can not only significantly improve the soil nutrient level but also positively increase the chances of its further invasion with more copious plant attributes Mandal et al., 2014. Due to fitness homeostasis, phenotypic plasticity, widespread geographical range, modes of reproduction and ultimately none and the most potent, the phenomenon of allelopathy *Lantana* is highly invasive Sharma et al., 2005. Some other allelochemicals identified in *Lantana camara* are cytotoxic in nature found in the leaves are lantadene A and lantadene B [17]. In forest due to allelopathy, *Lantana* density increases due to which species richness declines [18]. Allelochemicals present in *Lantana* hampers the vigourness of native plants of a particular region and ultimately results into poor productivity [19]. Wild fire in many forest rich parts of India is also caused due to *Lantana* invasion [10]. The stem, leaf and fruit leachates of *Lantana* inhibit seed germination and seedling growth of some terrestrial plants [20]. Due to high regeneration potential and sexual reproduction throughout the year favors their quick colonization in the area of their penetration [3]. The management of *Lantana* expansion in forest and cultivable lands is a big challenge for scientific community and policy maker Suthar et al., 2013. The allelochemicals present in *Lantana camara* inhibit the process of seed and spore germination evident from the research on Seeds imbibed in aqueous extracts of leaf, stem and root of *Lantana camara* [21]. Many species of *Lantana* are native to Africa and America and has covered many of the neighboring countries [18].

#### 2.1.2 Ageratum conyzoides

*Ageratum conyzoides* is also known as billy goat weed or goat weed. It belongs to the plant family Asteraceae. It is widely distributed tropical and subtropical areas of the world. Commonly found in cultivated areas, pasture land, road sides interfering with native plants. The plant is now found as a weed of over 36 crops (including plantations) in 46different countries [22]. It has been ranked as 19th of the world's worst weeds [22]. *Ageratum conyzoides,* a native of Central America and the Caribbean, is now found throughout the world [23]. The special feature of *Asteraceae* plant family is that the flowers massed together in a head, a special kind of inflorescence having advantage to attract pollinators for cross-pollination that helps in increase in population in a short period of time. Also effective seed dispersal mechanism by parachute like special organ developed on the fruit helps in long distance dispersal of the seeds. The large seed producing capacity helps this weed for rapid colonization and thus it invades easily. It produces a large number (8000–10000/plant) of fruits (achene) that have a pappus. These are easily disseminated by air, water and animals. The seeds are photoblastic and remain viable for one year [11]. These morphological characteristics are advantage for easy dispersion of this plant species that leads to rapid invasion. Absence of natural predators and high reproductive potential attributes to successful invasion. The yield of staple crops like rice, wheat, corn etc. has been reported to be diminished due to invasion of *Ageratum.* When it invades rangeland areas, it out competes native grasses causing scarcity of fodder [11]. Due to hindrance in field practices like ploughing, maintenance cost of croplands is increased by the growth of these weeds [11]. In the lower Shivalik range of the Himalayas due to infestation of cultivated lands by this weed to a great extent that led to leaving of farmers their agricultural fields. A wide range of chemical compounds including alkaloids, flavonoids, chromenes, benzofurans and terpenoids have been isolated from this species. Extracts and metabolites from this plant have been found to possess pharmacological and insecticidal activities [24]. Both the volatile oil and the aqueous extract of the *A. conyzoides* have been shown to have allelopathic effects on a number of cultivated crops. Through volatilizing, leaching, and residue decomposion into the environment many kinds of allelochemicals are released by A conyzoides. The saturated aqueous solution of the isolated and purified precocene I and II have been reported to have significant inhibitory effect on the seedling growth of radish, tomato and ryegrass [25]. Its allelopathic potential varied with growth stages and environmental conditions. It releases more volatile allelochemicals (ageratochromene and its derivatives, monoterpenes, sesquiterpenes and flavones) under adverse conditions [25].

#### 2.1.3 Parthenium hysterophorus

**Fig. 2:**
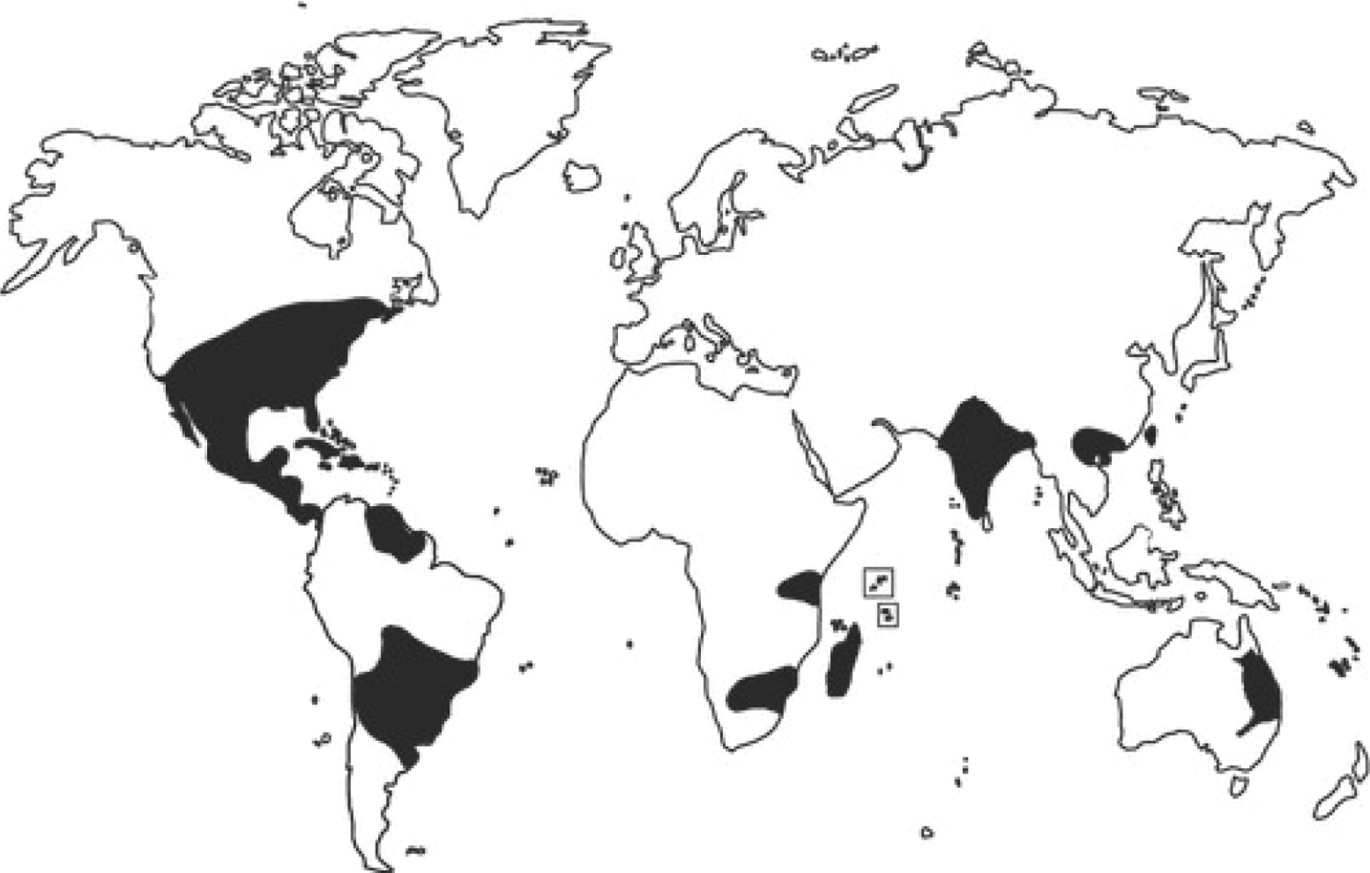
This figure was reported by [42]. Black color area represent the distribution of *P. hysterophorous.*

*Parthenium hysterophorus* is considered as one of the worst weed of the world. The plant belongs to the plant family Asteraceae, commonly known as Congress Grass. Native to southern United States, Mexico and Central and South America, it has been accidentally introduced into several countries and has become a serious agricultural and rangeland weed in parts of Australia, Asia, Africa and the Pacific Islands, as presented in Fig. 2. Almost every part of India this weed is prevailing. Wide adaptability, photo- and thermo-insensitivity, drought tolerance, strong competition and allelopathy, high seed production ability, longevity of seeds in soil seed banks, and small and light seeds that are capable of long distance travel via wind, water, birds, vehicles, farm machinery and other animal traffic, contribute to its rapid introduction world-wide, cutting across national boundaries and climate barriers. Due to its high fecundity a single plant can produce 10,000 to 15,000 viable seeds and these seeds can disperse and germinate to cover large areas. The allelopathic effect reduces crop production drastically. The biodiversity also threatened by aggressive dominance by this noxious weed. Many allergic respiratory problems, contact dermatitis, mutagenicity in human and livestock are reported to be caused by this weed [26]. The allelopathic effect of this weed is evident by various experiments carried out over the time. *Parthenium* weed is capable of interfering with the growth of neighboring seedlings even at very early stages of growth. *Parthenium* extracts contain allelopathic effect which could affect the seed germination and elongation of Onion and Bean demonstrated by an experiment carried out to evaluate the effect *of P. hysterophorus* on germination and elongation of Onion *(Allium cepa)* and Bean *(Phaseolus vulgaris)* [27]. A study carried out on the effect of *Parthenium* root extract on the germination and shoot growth of Maize and Barley it was reported that as the concentration of root extract increases, there is a decreasing trend in germination and shoot growth. On germination, shoot length, root length, seed vigour, tolerance index, root length, shoot length, fresh and dry weight of bean seedlings are inhibited by the *Parthenium hysterophorus* leaf, stem and flower extracts [28]. The aqueous extracts of root and shoot of *Parthenium* in higher concentration reduced the germination percentage and growth of Glycine max (soyabean). The productivity of this crop is adversely threatened by the *Parthenium* weed invasion (Netsere et al., 2011). *Parthenium hysterophorus* is a weed of worldwide impact [29], it presents over 20 countries in Africa, Asia and Oceania [30]; Fig. 2). On the African continent, *P. hysterophorus* arise from South Africa [[31], [32]]. Therefore, proper management with integrated approach is a major concern to control this invasive weed species.

#### 2.1.4 Saccharum spontaneum

**Fig. 3:**
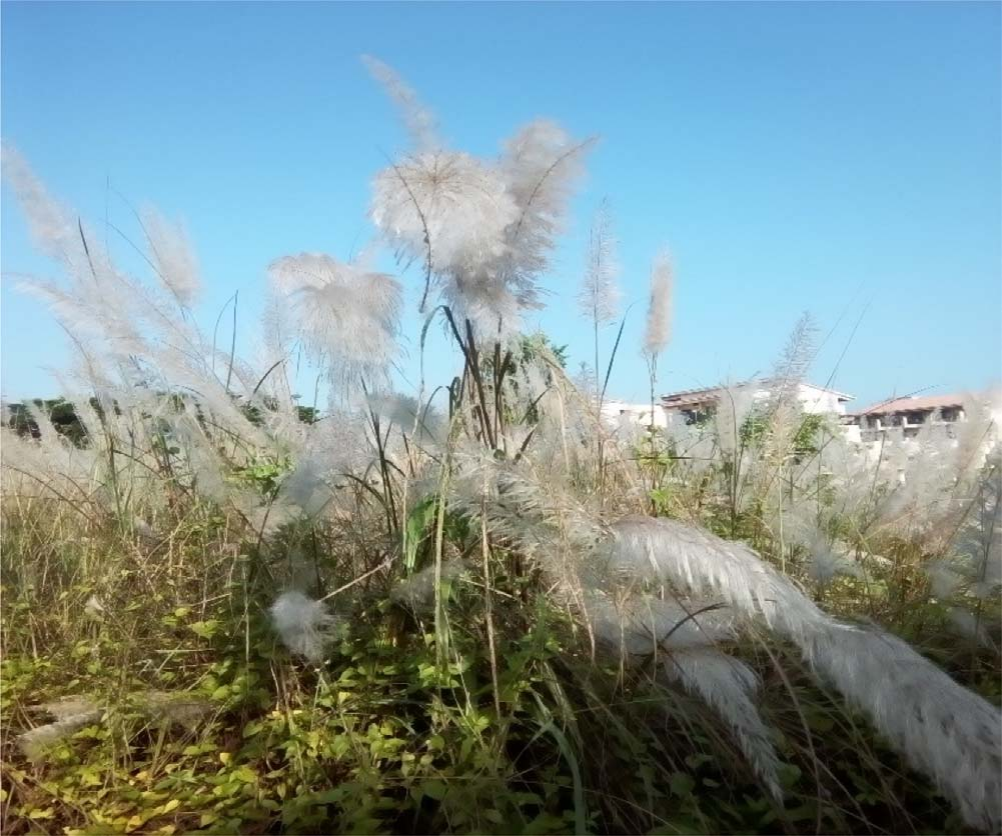
*S. spontaeneum*.

*Saccharum spontaneum* is a perennial, herbaceous plant belonging to the family Poaceace. This is a close relative and one of the most important parents in the interspecific hybridization of sugarcane as shown in Fig. 3. It has adapted to live over a wide range of grassland climatic habitats: from oriental Asia to the southern region, in the warm-temperate areas of Africa and in Mediterranean regions. In the Terai-Duar savanna and grasslands, a lowland ecoregion at the base of the Himalaya range in Nepal, India, Bangladesh and Bhutan, this grass quickly colonises in the exposed silt plains created each year by the retreating monsoon floods, forming almost pure stands on the lowest portions of the floodplain. The grasslands exhibited by *Saccharum spontaneum* are an important habitat for the Indian rhinoceros *(Rhinoceros unicornis)*. The productivity of many crop species has been reported to be reduced due to invasion of this species. Apart from that this is a host for many of the pests which spreads to the adjacent cropland. *S. spontaneum* is a serious weed of cotton, pearl millet, sorghum, sugarcane, rice, forage crops, horticultural gardens and plantation crops such as tea and coffee in tropical and subtropical climates. In India, germination and emergence occurs in June/July after the first showers of the rainy season; adult plants bear flowers by the end of the rainy season, as shown in Fig. 3. Reproductive potential about this plant is very less known. Mostly it is by means of rhizomes vegetative propagated and also produces large number of seeds. Seed dispersal to a large extent drives the spread of the species. Reforestation has been suggested as a strategy to control *Saccharum spontaneum*, an invasive grass that impedes regeneration in disturbed areas of the Panama Canal Watershed (PCW). *Saccharum spontaneum* distubes forest ecosystem by promoting fire and the natural regeneration is inhibited by this vegetation [33]. Height and competition of *S. spontaneum* correlated negatively with intensity of mechanical cleanings and herbicide application. The cost of herbicide application and mechanical cleaning is significantly increased [34]. Apart from negative adversity of *Saccharum spontaneum* it has a potential for second generation bioethanol production. *Saccharum spontaneum* might be introduced into the Mediterranean cropping systems to supply lignocelluloses biomass for second-generation industrial plants or bio-refineries due to high levels of biomass yield and composition of structural polysaccharides [35].

### 2.2 The different weed management practices

There is multiple prevention and elimination strategies are adopted. These are: cultural, mechanical, biological, Chemical etc. Different cultural practices mulching, using transplants, tillage, drip irrigation, rapid cleanup after harvest etc. reduces weed numbers but cannot eliminate weed completely. Stale seedbed techniques are to prepare soil for planting and bring weed seeds to the surface, allow weeds to germinate and kills weeds with light tillage. But after this method also there will be residual amount of weed biomass. Cover crops may reduce weed emergence by 75% −90% but still there is remnant of weeds. Weeds tend to infest crops having the similar life cycle. Changing cultural practices related to cultivation process, use of fertilizers, application of herbicide may lead to management of weed but to eradicate weeds completely is beyond possible. Physical and mechanical practices also used like mourning, cultivation, soil solarization, Hand weeding, Flaming. But these processes have cost-benefit impact. There are various adverse effects of all these processes. Cultivation is a practice better for perennial and biennial control than annual weed control. Some of the processes require month long treatment processes. The different drawbacks of cultivation processes are it exposes the bare ground, leads to soil compaction, cost expensive equipment’s are required, cannot be processed in wet condition. Flaming is another process to knock down weed seedlings prior to planting and just before crop germination flaming used to kill weed seedlings. But all of these processes lead to the generation of weed biomass which is ultimately going to the waste stream and there is no proper method for the disposal of these weeds. Biological control is also practiced in various time. California's most pervasive weed yellow star thistle have been reported to be controlled by six species of overseas insects [36]. As conventional weed management practices are unsuccessful for the weed lythrum salicaria, a biological control by root boring weevil *Hylobius transversovittatus* (highly host specific to the target weed have been approved in 1992 [37]. Though biological control attempts have been made on *L.camara* longer than on any other weed, the plant is still not under adequate control. As it is a hybrid species consisting of many phenotypes, originating from two or more species of *Lantana* in tropical America, and that it grows in a wide range of climatic areas that influences the biocontrol of this plant [38]. But biocontrol of weed can cause serious problem due to attack by agent on non-target plant species and therefore it is essential to protect the non-target species. Host specificity is required before approving release of agents. There is various evidence in history of unforeseen interaction of agent with non-target plants. The chemicals methods used to kill weeds using various weedicides have various environmental consequences. The soil fertility is lost due to repeated application of chemicals and also using these chemicals in agricultural fields, the trances of these chemicals enters the food chain. The results of hand weeding are significantly better but as it is time consuming and laborious to human, hence it cannot be recommended at large scale [39]. The application of herbicides is common today and is used in wide scale. But concern is directed towards its impact on non-target organisms as well as an ultimate fate of these chemicals on our environment. Even finding small traces of these elements on our environment is a major threat. The residual of these elements in entire ecosystem leads to unsustainable condition specially the aquatic system where the drainage flows from areas treated with herbicides. The prevention of water contamination is the utmost concern for human kind and for the whole ecosystem. There are various data regarding toxicity of weedicides to fish are available [40].

### 2.3 Vermicomposting, an alternative viable option

The experiments regarding vermicomposting of various terrestrial weeds are carried out at different time. Many of these weeds results into preparation of good quality compost inferred by different researchers. The experiments were conducted to obtain compost from some toxic weeds by using vermicomposting and conventional methods. The weeds used in the experiment were congress grass *(Parthenium hysterophorus*), water hyacinth (*Eichhornia crassipes*) and bhang *(Cannabis sativa Linn.).* Total six sets of experiments were setup by using above materials. The results show a high increase in nitrogen, potassium, phosphorus and a high decrease in organic carbon, C/N, C/P ratio in the experiment having *Eisenia fetida* [41]. There some work has been done on Vermicomposting of terrestrial weeds reported on Table 1.

**Table 1:** Work done on vermicomposting and composting of different terrestrial weeds.

Author Year	Work done
Biradra et al., 2001	Vermicomposting of *Pathenium hystrophorous*
Sivakumar et al. [43]	Efficiency of composting Parthenium plant and Neem leaves in the present and absent of an olligochaete Eisenia fedita
Chauhan and Joshi [41]	Compost of some toxic weeds by vermicomposting and conventional methods.
Kishor et al. [44]	Vermicomposting with Eisenia fedita of *Parthenium hysterophorous* mixed with cow dung with different ratio (25, 50, and 75%)
Anbalagan and Manivannan [45]	Vermicomposting of *Parthenium hysterophorous*
Reddy et al., 2012	Germenitation of tomato seeds was observed in *Parthenium* and sludge mediated vermin extract.
Sivaraj et al., 2013	Composting and vermicomposting of *Parthenium hysterophorous*
Suther et al., 2013	Germination index was determining with *Lantana camara* mediated vermicompost.
Rajiv et at., 2013	Germination Index was determined with *Lantana camara* mediated Vermi compost.
Mistry et al. [46]	Nutrient value of vermicompost from *Perthenium hysterophorous* and *Argemone Mexicana* was analysed.

Vermicomposting with *Eisenia fetida* of *Parthenium hysterophorus* mixed with cow dung in different ratios (25%, 50% and 75%) in an 18 weeks’ experiment shows that in all the treatments, a decrease in pH and C: N ratio, but increase in EC, N total, P available, Ca total, K total and heavy metals was recorded. The cocoons production and growth rate were maximum in 100% cow dung. The results indicated that *Parthenium* can be a raw material for vermicomposting if mix with cow dung in appropriate quantity [47]. The macronutrients (N, P and K) and micro nutrients were increased in *Parthenium* mediated vermicompost and decreased in compost of *Parthenium* mediated compost. *Parthenium* can be utilized effectively as organic manure by composting and vermicomposting and thereby control this weed [48]. The leaves of Neem and *Parthenium* were composted in experimental set-up with Earthworm and without worm The result shows that higher *Parthenium* concentration reduced the growth and reproduction of *Eisenia fetida* (Earthworm species). Among *Parthenium* compost and neem compost significant difference were not observed [49]. In various experiments *Lantana camara* has beeen used by researchers for the production of vermicompost. The germination index (GI) was between 45% and 83% in all vermicompost as indicated by the seed bioassay test. The suitability of some weed species in vermicomposting production was evaluated in an experiment conducted in Bijapur, Karnataka. *Parthenium hysterophorus* was reported to be superior substrate for vermicomposting as in clitellate and non-clitellate worm’s biomass were higher per bed compared to other weeds [50]. The *Azotobacter* microflora reported to be more in *Parthenium hysterophorus* mediated vermicomposting. The yield of vermicompost is more, predicted due to presence of some important microbes that help the earthworms to act faster on the weeds [51]. Composting of uprooted *Parthenium* can be a good option to stop its invasion and inhibit the growth of the weed. The high component of essential elements in compost from *Parthenium* increases the crop yield [44]. It was observed that *Parthenium hysterophorus* mixed with other organic supplements imparts suitable physico-chemical conditions for maximum worm production and large scale vermicompost production. The future research is required to explore the utilization of *Parthenium hysterophorus* in vermicompost production to enhance crop yield [45]. *Parthenium hysterophorus* can be used as additive for effective vermicomposting process of sludge. Maximum rate of 80% germination of tomato seeds was observed in *Parthenium* and sludge mediated vermin extract. The combination of *Parthenium* and cow dung enhanced the nutrient value of the compost and increased the germination of *Arachis hupogeae* the toxicity of allelochemicals could be minimized though compost [52]. When *Parthenium* mediated vermicompost was analyzed with vermicompost produced from another weed *Argemone Mexicana*, the higher concentration of nitrogen was seen in *Parthenium* vermicompost than other weed. Overall NPK value is also observed in higher concentration in both the weeds [46]. Various physical, chemical and biochemical characteristics that were tested, the vermicomposting seemed to be plant-friendly, giving finest results after applied at concentrations of 1.5% in soil (w/w) [53]. In the similar way the experiments can be carried out with other terrestrial weeds which are major constituents of agricultural land and also available in other land cover. The significance of vermicompost prepared by these terrestrial weeds can be analyzed and can be initiated for a large scale. There are so many terrestrial weeds and dominating invasive species. The compost prepared out of it can be used as organic fertilizer and soil conditioner.

The initial characterization of the weeds is reported in Table 2. Adequate moisture content is the major requirement for efficient vermicompost by the worms. Basically moisture level higher than 50% is required. In the initial characterization for all the plant weed substrates the moisture level is between 70% to 80%. The C: N ratio is also in the range of 25 to 30 for all the plant substrate is suitable for the rapid degradation by the earthworms and makes an excellent environment [54].

**Table 2:** Initial characteristics of the weeds.

Initial Characterization	*A. conyzoides*	*S. spontaeneum*	*L. camara*	*P. hysterophorous*
BOD(mg/L)	630	1050	750	890
COD(mg/L)	3000	3720	3552	3108
pH	6.08	6.9	6.73	6.75
EC(mS/cm)	5.3	5.61	5.1	5.32
Moisture Content %	75.63	75.22	75.83	73.77
% Volatile Solids	71.6	87.2	73.3	83.13
Ash Content	28.4	12.8	27.1	19.6
% Total organic carbon	41.53	50.55	44.32	45.33
TKN %	1.43	1.8	1.6	1.67
C:N ratio	29.04	28	27.7	25.05

## 3 Anaerobic digestion, a possible management option

Anaerobic digestion is another way to managed this noxious weeds and it also sustainable conversion of energy, where exhaust of fossil fuel is one of the key issue. They’re very few work have been done related to anaerobic digestion of terrestrial weeds. Anaerobic digestion is the process of biogas production, where particulate organic matters dissolved in soluble forms with the assistance of robust, mixed culture microbial communities in absent of oxygen; aiding in the conversion process of waste to energy [55]. The substrates which contain high moisture or semi solid are preferred for anaerobic digestion [56]. Increase of VFA can lead to drop in pH inhibiting the growth of microorganism and high ammonia concentration is toxic for anaerobic bacteria [57]. Higher the COD recovery higher the biogas. Acidic pH can be maintained neutral by addition of inoculums [58]. *Parthenium* can be done alternative feed stock for anaerobic digestion to produced methane [59]. *Parthenium* have the potential to produce to produced alcoholic biofuels after pretreatment [49]. *Parthenium* content 23% lignin of its volatile solid so pretreatment is necessary to increase the biogas production [59]. Pretreatment makes the substrate easier to microorganism for degrade and it enhance the biogas production [60]. Inoculum is the source of nutrients that boosts the enzyme activity leading to higher substrate degradation and biogas production [61]. Manure of lives stock holds high content of nitrogen like chicken manure (1.03%), fresh goat manure (1.01%), dairy manure (0.35%) and swine manure (0.24%) [62]. So when inoculums used along there is chances of ammonia toxicity in anaerobic digestion [63]. To maintains proper C/N ratio inoculums are mixed with substrate together yield of biogas is higher. Over all dairy manure shows the best result [64]. *L. camara* is practice for ethanol production is suggested by Sharma et al. [19]. There not much work has been done in anaerobic digestion of *Parthenium hysterophorous* as well as Other weeds like *A.conyzoids*, *S.spontaneum, L.camara*, Initial characterization shows this noxious weeds have the potential to produced biogas as there high moisture contain and pH is nearest to 7 as shown in the Table 2. Soluble chemical oxygen demand is also high which is also benefit for the methane generation [60]. It has been studied in small scale but large scale study has not been done. All though in smaller scale it shows 59% methane production. So more research is necessity for digestion of *Parthenium hysterophorous, A.conyzoids, S.spoutaneum and L.camara in* continuous and pilot scale.

## 4 Adverse effect on crops

*Parthenium* competition increased with increased its dry mass up to 448% and relative competition index up to 52% and consistent increases in the uptake of N (up to 581%), P (up to 700%) and K (up to 669%) were also recorded, competition period of *Parthenium* weed competition period is 35 DAE decreased of grain yield and produce index of maize [65]. Over the competitive displacement, it can cause up to 90% degeneration in the herbaceous constituent of natural plant communities where this weed invades [66]. As the growth of *Parthenium* increased NPK-uptake was also increased, so this weed-crop competition with agriculture crops, it was observed that uptake of *Parthenium* during the different competition period at the range of 2.7–18.4, 0.2–2.4 and 2.3–17.7 N, P and K kg ha^−1^, respectively at its different competition periods [65]*. Ageratum conyzoids* species has been informed to be one of the most overriding weeds of upland crops throughout South-east Asia. *Lantana camara* is a major problematic in agricultural zones in most of the states in India as it forms dense thickets, spread openly, and also disturbs equally flora and fauna [67]. *Lantana camara* have been verified that it damaged the growth of various crops like various crops like wheat (Triticum aestivum), corn (Zea mays) and soyabean (Glycine max) [68]. *Lantana camara* inhabits the land at disbursement of several other kinds of plants [53]. Research gaps occur for humid forest zones which are now an over-all combination of primary forest and secondary vegetation scattered with covers cleared for cultivation or other non-forest practices [69]. All though not much research had been done yet but more research is required to convert this notorious weeds into valuable product.

## 5 Conclusion

Rapid spreading of this terrestrial weeds is threatening the world mostly agricultural land. The management of these weeds had been tried into various way like manually, mechanically and burned. Weedicides was applied to the field to remove the weeds. But this kind of management is not able to provide sustainable and economic benefits. Using weedicide may reduce the soil fertility and also caused environmental pollution. More research is required to manage this noxious weeds to save the agricultural land and ecosystem. Anaerobic digestion is the sustainable route for the conversion of terrestrial weeds into biogas, where energy is one of significant issue in the world, other than that vermicomposting is the another way of control these weeds where it converts into valuable nutrients which can be applied into the agricultural field.

## Conflict of interest

With due respect and humble submission I would like to state that the authors have no conflict of interest in publishing the review paper “**Vermicomposting and anaerobic digestion- viable alternative optionsfor terrestrialweed management − a review”** in your esteemed journal.
